# Neonatal Immune Adaptation of the Gut and Its Role during Infections

**DOI:** 10.1155/2013/270301

**Published:** 2013-05-02

**Authors:** Emilie Tourneur, Cecilia Chassin

**Affiliations:** ATIP-Avenir Group, INSERM U699, Université Paris Denis Diderot, Sorbonne Paris Cité, Site Xavier Bichat, 75018 Paris, France

## Abstract

The intestinal tract is engaged in a relationship with a dense and complex microbial ecosystem, the microbiota. The establishment of this symbiosis is essential for host physiology, metabolism, and immune homeostasis. Because newborns are essentially sterile, the first exposure to microorganisms and environmental endotoxins during the neonatal period is followed by a crucial sequence of active events leading to immune tolerance and homeostasis. Contact with potent immunostimulatory molecules starts immediately at birth, and the discrimination between commensal bacteria and invading pathogens is essential to avoid an inappropriate immune stimulation and/or host infection. The dysregulation of these tight interactions between host and microbiota can be responsible for important health disorders, including inflammation and sepsis. This review summarizes the molecular events leading to the establishment of postnatal immune tolerance and how pathogens can avoid host immunity and induce neonatal infections and sepsis.

## 1. Introduction

Harboring trillions of microbes, the intestinal mucosa represents a complex ecosystem playing a dual role in host defense. Permanently exposed to enteric microbes, the mucosa has to provide an efficient protection against pathogenic microbes, and, on the other side, has to maintain tolerance towards commensal flora. The innate immune system has evolved to provide mutual profit to both the host and microbiota. Commensal bacteria, expressing unique enzymes, contribute to the digestion of dietary substances as well as the synthesis of food supplements [1]. They also confer protection against pathogenic bacteria though competition for space and nutriments [2, 3]. Commensal flora induces innate immune signaling which favors the differentiation and maturation of the immune system, the maintenance of the barrier integrity, and restricts commensal flora to the lumen [4–8]. On the other hand, the innate immune system has to be controlled and the intestinal mucosa develops mechanisms of tolerance that enable microflora to thrive and mechanisms of defense to provide an efficient response in case of invasion by pathogens. This subtle balance of the innate immune signaling is tightly controlled, and disturbance of this host-commensal relationship may cause inappropriate response of the innate immune system leading to inflammation, organ dysfunction, infections, sepsis, or cancer [9–11]. 

**Figure 1:**
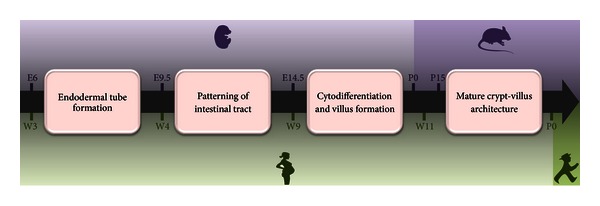
Time line of intestinal development in human and mice. Definitive endodermal cells are specified during gastrulation (mouse: embryonic day 6 E6, human: week 3 W3) and initiate the formation of the primitive gut tube, fully formed at E9.5 in mice and W4 in humans. At later stages, the tube is patterned along the anterior-posterior axis. Cytodifferentiation and villus formation take place from E14.5 in mouse and from W9 in human. In mice, crypt formation starts around day 15 after birth (postnatal day 15, P15), whereas in humans, mature crypt-villus architecture is already defined during fetal period from W11.

The intestinal surface is covered by a monolayer of polarized epithelial cells which, from birth to death, represents the only border separating the microbes of the intestinal lumen from the host. The challenge is particularly complex since the essential function of gut is to exchange nutriments with the content of the lumen, representing the major part of body nutrition. Conversely, the direct contact between the intestinal bacteria and the epithelial cell surface has to be minimized and controlled to avoid an inappropriate activation of the immune system. During ontogeny, the formation of the primitive gut starts early and is initiated from cells of the endoderm [12–14]. In mice at E6, definitive endodermal cells are specified during gastrulation. At E8.5, the endodermal tube is initiated by the fold of the endodermal lining at the anterior and posterior ends, creating anterior and caudal intestinal portals. After gut tube formation at E9–9.5, the simple epithelium turns into a pseudostratified epithelium. Specific intestinal markers, such as villin, first appear in the hindgut at E9 [15]. Between E9.5–14.5, while the gut length and circumference increase, the primitive gut tube is patterned along the anterior-posterior axis. A transitional period in the course of which the epithelium turn stratified was thought to occur, but a recent study shows that this event may not take place [16]. Around E14.5–15, gut epithelium begins a remodeling process with the emergence of finger-like protrusions called villi on the previously flat luminal surface, providing efficient nutrient absorption. Of note, unlike the small intestine, villi are lost during fetal development of the colon mucosa. Cell proliferation, firstly homogenous along the epithelium, becomes limited to the intervillus regions where gland-like invaginations (named crypts) secondarily start to form, creating a protected stem cell niche. These groups of stem cells migrate in a crypt-villus axis and are behind the different cell phenotypes of the intestinal epithelium. The level of maturity of neonatal gut at birth differs between species and depends on the length of the gestation period (Figure 1). Whereas human and guinea pig small intestine presents mature crypt-villus architecture at birth, crypts emerge 12–15 days after birth in mice during the weaning period [17]. In humans, the fetal gut is structurally mature from week 19 of gestation, and all the cellular components of the gastrointestinal immune system are already present during the fetal life. For example, T cells are identified around 12 weeks of gestation [18]. Nevertheless, the gastrointestinal immune system remains immature at birth, since antigenic stimulation of the colonizing microflora is required for its full maturation. 

Cytodifferentiation goes along the villus/crypt axis formation. The immature primitive stem cells localized in the crypts lead to the formation of distinct lineages of intestinal epithelial cells based on their functions: enterocytes, goblet cells, enteroendocrine cells, and Paneth cells [19]. Notch-mediated signaling pathway triggers epithelial cell differentiation and is essential for gut homeostasis [20]. Enterocytes are absorptive cells which represent 90% of intestinal epithelial cells. The apical surface is lined by a microvilli-covered brush border where essential enzymes and transporters for nutrition are expressed. Secretory cells are divided in three types: goblet cells, enteroendocrine cells, and Paneth cells. Goblet cells are the most abundant secretory lineage in the gut epithelia and are involved in the production of highly glycosylated mucins generating a mucus matrix acting as a protective barrier by covering the gut mucosa [21]. It has been suggested that they could participate in the delivery of luminal antigens to subepithelial antigen-presenting cells [22]. They are located throughout the epithelial surface and their number increases from the proximal small intestine to the colon. Enteroendocrine cells are divided in more than 16 subtypes identified in mouse intestine depending on hormones or other signal mediators secreted. Their role as immune sensors is still unclear, even though it has been shown that they express a variety of innate immune receptors and respond to microbial stimulation [23]. Paneth cells, located at the bottom of the crypt of the small intestine, produce and secrete antimicrobial peptides and soluble mediators in the lumen, creating a niche for stem cells and reinforcing the mucus layer. Of note, they are lacking in the colon, as well as in the intestine of some species such as *Xenodon merremii* [24]. Stem cells allow a constant renewal of gut epithelium which must be maintained throughout the course of life. Transit-amplifying cells, after about two days in the crypt, divide 4-5 times before being terminally differentiated into one of the specialized intestinal epithelial cell types. In adult mice, around three days after the end of their differentiation, the cells reach the top of the villus, enter in apoptosis, and are exfoliated to the gut lumen [25]. The different cell types of the epithelium appear at different times during gut formation. In mice, Paneth cells appear after birth during the emergence of crypts in the small intestine whereas enteroendocrine cells are already present around E10. After birth, cell proliferation is low in the intestine of neonate mice until weaning in correlation with suckling diet and increases around 10–12 days after adaptation of the gut epithelium to solid nutrient components [26, 27]. Importantly, transcriptional repressor B lymphocyte-induced maturation protein 1 (Blimp1) is highly expressed in the developing and postnatal intestinal epithelium until the suckling to weaning transition. It has been shown that this factor is accountable for the developmental switch responsible for postnatal intestinal maturation and governs the suckling to weaning transition of the epithelium [28, 29]. The generation of new mouse models, such as the multicolor Cre-reporter R26R-Confetti mice, will probably bring new insights in the development and maturation of the intestine [30]. 

This review focuses on the major mechanisms and factors that are crucial for the establishment of the immune intestinal tolerance in the first weeks after birth, as well as the maintenance of a life-time homeostasis. Finally, the postnatal dysregulations of these processes possibly leading to infant inflammatory diseases such as neonatal infections and sepsis will be addressed.

## 2. Postnatal Colonization of the Gut

In normal conditions, fetal gastrointestinal tract is thought to be sterile. However, studies have suggested that fetal gut can be exposed to microorganisms invading the amniotic fluid, which can be associated with preterm delivery [31, 32]. It is also well known that prenatal exposure of the mother to bacterial components can influence intestinal epithelial development and function in newborn, as well as sensitivity to inflammatory diseases such as necrotizing enterocolitis [33–35]. Thus, prenatal exposure of the gut to bacteria may modulate immediate postnatal adaptations inducing tolerance toward colonizing bacteria. During birth, the intestinal mucosa undergoes a dramatic transition from a protected site to a densely colonized environment [36, 37]. Delivery allows the first contact between gut epithelium and microorganisms. Newborns are mainly exposed to microorganisms from the maternal mucosa and endotoxins of the environment. In mice and humans, after birth, facultative anaerobic or microaerophilic bacteria such as *Lactobacilli* and *Streptococci* are dominant. Few days later, *Enterococci* and Enterobacteriaceae appear and generate a decrease of local oxygen concentration by their metabolic activities, favoring the colonization by *Bifidobacteria*, *Bacteroides* spp. and *Clostridium *spp. [38–40]. Less is known about the longitudinal pattern of colonization after birth and the differences along the length of the gastrointestinal tract. Interestingly, transcriptome analyses show significant spatial differences in the response to colonizing microflora in the jejunum and colon compared to the ileal segment [41]. 

The colonization of the gut is influenced by several factors. The first microbial exposure of the newborn, as well as the delivery mode (i.e., vaginal delivery versus caesarian-section) has been shown to influence the postnatal gut microbiota composition [42]. Associations between some specific constituents of microbiota in human newborn day 4 after birth and the concentration of specific microbial groups at day 120 have been demonstrated, suggesting that early gut microbiota may influence later microbiota [43]. Colonization of the gut of caesarian-section-born infants appears to be delayed compared to vaginal delivery born infants. Moreover, caesarian-section born human babies have a different colonization pattern compared to vaginal delivery born ones [44]. However, vaginal microbes of the mother seem to not settle in neonate gut and change rapidly with suckling [45]. Although neonatal rodents are exposed to greater numbers of environmental microbes than humans, similar findings have been shown in culture-based studies demonstrating that the initial flora of the neonates is mainly composed of the vaginal and fecal microorganisms of maternal origin [46]. Diet (i.e., breast milk versus formula) is also a factor influencing the composition of the microflora. *Bifidobacteria *is dominant in the microflora of breast milk fed neonates, whereas formula-fed infants microflora harbor a majority of *Bifidobacteria*, *Bacteroides* spp., and *Clostridium *spp. [47]. Under the influence of the diet (i.e., milk to solid food) and especially after weaning, the composition of the microbiota changes rapidly. Gut microflora has fully matured in children around the age of 4 and after 3 to 5 weeks in mice [37, 48] and remains relatively stable throughout life [49, 50]. Other factors, such as hygiene, environment, and lifestyle, also influence the initial composition of the microflora, but at a lower extend once the microflora is stabilized. Indeed, the microflora of marital partners does not have a significantly greater similarity in their composition than unrelated individuals, even if these partners live in the same environment and have similar dietary habits [51]. Besides, the microbiota of monozygotic twins living separately is notably more similar than the microbiota of unrelated individuals [52].

The mature microbiota contains a complex and dynamic population of more than 1000 different microbial species in the human gastrointestinal tract, reaching 10^12^ cfu/g of gut contents in the large bowel [53]. The collective genome of the whole microbes, the microbiome, may contain more than 100 times the number of genes of the mammalian genome [54]. Gut microbiota is increasingly considered as a “metabolic organ” inside the gut intestinal tract, which acts in physiology to develop functions that humans have not evolved for their own. The impact of the microbiota on gut physiology, metabolism, and health has been shown to be largely influenced by microbial activities, like fermentation of food components not digested by the upper gastrointestinal tract such as nondigestive carbonate [55]. Interaction between host and bacteria in the gut mucosa is, of course, essential for host digestive efficiency and intestinal physiology, and plays a major role in the establishment of immune postnatal tolerance after birth, as well as in the maturation of the gut-associated lymphoid tissue [27, 56, 57]. Doubtlessly, the microbiota and the immune system of the host interact in a two-way street: while bacteria induce immune maturation, the host immune system regulates the number and the composition of the bacteria. 

## 3. Adaptation of the Gut Mucosa to Bacterial Colonization

### 3.1. Crosstalk between Bacteria and Host Cells

The first exposure of the gut to bacterial ligands occurs during the passage though the birth canal or shortly thereafter. This interaction between colonizing bacteria and host cells has been shown to be crucial for the establishment of intestinal tolerance (Figure 1). Bacterial ligands are recognized by innate immune receptors, such as Toll-like receptors (TLRs), which are expressed by intestinal epithelial cells throughout fetal, neonatal, and adult life. Both TLR2 and TLR4 are expressed in human fetal tissue from 18 weeks of gestation [58]. TLR1-5/9 and TLR1-9 have been detected in human small intestinal tissue and colon, respectively and TLR1-9 and TLR1-4/9 in murine small intestinal tissue and colon respectively [59]. Intestinal epithelial cells also express the cytosolic helicases retinoic acid-inducible gene I Rig-I and melanoma differentiation-associated gene 5 Mda5, sensing the presence of RNA and Nod-like receptors such as Nod1 and Nod2, sensing the DAP-type tripeptide motif or the muramyl-di-peptide motif of peptidoglycan, respectively. Epithelial Nod1 and Nod2 seem to synergistically protect the colon during *Salmonella enterica* subsp. *enterica* sv. Typhimurium (abbreviated *Salmonella *Typhimurium) infections [60].

Interestingly, we showed that the postnatal exposure of epithelial cells to lipopolysaccharide (LPS), the endotoxin found in the outer membrane of Gram-negative bacteria which activates TLR4, drives the neonatal innate immune tolerance in epithelial cells [27, 38]. This state renders epithelial cells hyporesponsive to TLR stimuli and protects the gut during the maturation of the mechanisms involved in adult intestine homeostasis. At birth, the first contact between the LPS of the bacteria and the epithelial TLR4 induces the production of microRNA (miR)-146a, a small noncoding RNA. miR-146a specifically inhibits the translation of the interleukin-1 receptor-associated kinase (IRAK) 1, an essential kinase of the TLR4 pathway. In other words, the activation of TLR4 during birth induces the activation of an inhibitory loop through the expression of miR-146a, shutting down the ability of the cell to respond to TLR agonist. This IRAK1^low^ state protects the gut during the first contact with bacteria, since the administration of an anti-miR-146a to neonates reestablishes the expression of IRAK1 in epithelial cells as well as TLR susceptibility and induces inflammation, apoptosis, and damages in the gut mucosa during bacterial colonization. On the contrary, the administration of a miR-146a mimic to caesarian-section born mice, which normally fail to decrease the epithelial IRAK1 level due to the lack of TLR4 activation in LPS-free conditions after birth and develop intestinal damages during colonization, rescues the intestinal phenotype. Moreover, the IRAK1^low^ state sustains the expression of specific genes that are important for cell maturation, survival and nutrient absorption. The switch to an IRAK1^high^ state progressively occurs after day 14, when cell proliferation increases, crypt-villus architecture appears and immune tolerance is acquired. The IRAK1^high^ epithelial cells are able to respond to LPS stimulation and are fully functional to activate an inflammatory response against pathogens. Strikingly, the IRAK1^low^ state in epithelial cells during the postnatal period seems to be maintained by an active constitutive signal mediated by internalized endotoxin. The loss of endotoxin is correlated to the increase of proliferation and allows the change from an IRAK1^low^ to an IRAK1^high^ state. Even though TLR signaling sensitivity is decreased in the neonatal intestinal epithelium, a number of other innate immune receptor signaling remain fully functional during this period. For example, the helicases Rig-I and Mda5 mediate the antiviral defense against rotavirus infection by promoting interferon *λ* production [61, 62].

Epithelial crosstalk with colonizing bacteria seems to be essential for immune tolerance since a specific epithelial lack of the proinflammatory transforming growth factor (TGF) *β*-activated kinase 1 (TAK1) leads to early inflammation, Tnf-dependent induction of apoptosis, tissue damages, and postnatal mortality [63]. Similarly, spontaneous mucosal damages have been observed during early postnatal development of mice specifically deleted in epithelial p65/RelA NF-*κ*B subunit. In inhibitor *κ*B kinase (Ikk) 1/2 (also known as Ikk-*α* and *β*) knockout and myeloid differentiation primary response gene 88 (Myd88) dominant-negative mice, translocation of commensal bacteria is increased and inflammation occurs due to a lack of homeostatic signaling. Accordingly, epithelial-specific deficiency of Nemo (also known as Ikk-*γ*) and Ikk1/2 in a Myd88 knockout background does not cause inflammation [64]. Clearly, an active dialogue between colonizing bacteria and epithelial cells exists and is essential for the maintenance of homeostasis in the gut. With the use of germ-free mice and the development of high-throughput sequencing, this concept has been extensively studied, highlighting that this interaction drives several aspects of innate and acquired immune response of the host [65, 66]. In germ-free mice, the absence of microbiota impairs the immune development of the intestine, and mice exhibit underdeveloped gut-associated lymphoid tissue including rudimentary Peyer's patches and intestinal follicles, reduced number of CD4^+^ T cells and immune effector molecules as wells as intraepithelial lymphocytes (IELs), decreased MCH class II expression on antigen-presenting cells, and decreased immunoglobulin A (IgA) production [67–70]. Reconstitution of the microbiota or even inoculation of specific bacterial components such as polysaccharide A (PSA) largely corrects these deficiencies and reconstructs aspects of the mucosal immune system [71]. Thus, the presence of the microbiota in the gut allows the development of an extensive and activated intestinal immune system. Recent findings have given some keys about the way the microflora influences the development of the immune system. After detection of the microorganisms by innate immune receptors, epithelial cells produce cytokines such as IL-10 and TGF-*β* which can regulate professional immune cells of the lamina propria. The balance between Th1, Th2, and Th17 responses has to be fine-tuned to maintain homeostasis. Particularly, CD4^+^CD25^+^ regulatory T (T_reg_) cells contribute to the maintenance of self-tolerance, suppress inflammatory response by secreting anti-inflammatory cytokines, and can inhibit the function of antigen-presenting cells. The activation of intestinal epithelial cells by some commensals such as *Clostridium *spp. induces the production of TGF-*β* which in turn allow the accumulation of IL-10-producing Foxp3^+^  T_reg_ cells [8]. Also, migratory CD103^+^ CCR7^+^ dendritic cells are conditioned by microbial and epithelial-derived factors and promote the differentiation of Foxp3^+^  T_reg_ cells as well as IgA production by B cells [72]. Epithelial cells also react to the alterations of the immune system, and the gene expression profile can switch from metabolic activities to epithelial host defense in the absence of IgA, for example [73]. Meanwhile, segmented filamentous bacteria (SFB) mediate Th17-cell as well as Th1 and T_reg_ population initiation, raising protection against bacterial infections [5]. Also, acetate from *Bifidobacterium longum* enhances intestinal barrier integrity through induction of antiinflammatory and antiapoptotic genes and protects mice from lethal infection with *Escherichia coli* O157:H7 [74]. The different mechanisms by which pathogens and commensals differentially activate the immune system of the host in the intestine remain partially understood and are extensively studied currently. It has been proposed that the immunologic distinction between pathogens and the microbiota is mediated by host mechanisms, and also *via* the recognition of specialized molecules evolved by symbiotic bacteria to enable commensal colonization, such as PSA from *Bacteroides fragilis* which activates TLR2 pathway on Foxp3^+^  T_reg_ to engender mucosal tolerance [75].

### 3.2. The Intestinal Mucus Layer as a Protective Barrier for the Host against Bacteria

The mucus barrier covering the intestinal mucosa forms a protective physical shield against bacteria and limits the microbial contacts with the host cells. The small intestine and the colon harbor different type of mucus layers. The small intestine is covered by a single layer of mucus mainly composed of the protein mucin 2 MUC2, released at the crypt openings [76]. Probably covering the villi, the mucus is unattached to the epithelium and is permeable to bacteria which can be trapped in the pores of the MUC2 network, bind the hydrophobic CysD mucin domains conferring stickiness to mucus, or bind the polymorphic and variable mucin glycans though their adhesins [76, 77]. By contrast to the small intestine for which a nonpermeable mucus layer would be detrimental for nutrition function, the colonic mucosa is covered by a dense and thick double layer of mucus [78]. The inner layer is attached to the epithelium and forms a compact bacteria-free coat, of around 50 *μ*m thickness in mouse and up to several hundred *μ*m in human. Secretions of goblet cells allow the inner mucus layer to be renewed and the upper part is converted to the loose permeable outer mucus layer which expands until 5 fold in volume due to endogenous protease activities acting on the MUC2 mucin, creating a habitat for the intestinal microbiota [79]. Bacteria utilize the released mucin monosaccharides as an energy source in addition to undigested carbohydrates from the food, thus producing short fatty acids able to diffuse through the inner mucus layer for the host. 

The mucus layer clearly plays a major role in host/microbiota homeostasis. In mice deficient in MUC2, bacterial contact with the host mucosa is increased, leading to the development of spontaneous colitis, and later on, colorectal cancer [80, 81]. As described before, commensal bacteria generally use the mucus and adhere to the mucus matrix. Some of them, such as SFB, penetrate the mucus and directly interact with epithelial cells for the maturation of mucosal specialized immune cells [5, 6]. Moreover, some pathogenic bacteria such as *Listeria monocytogenes* and *Salmonella *Typhimurium developed the ability to penetrate the mucus matrix to invade the epithelium by targeting goblet cells specifically [82, 83].

At birth, the production of mucin is low in the gut, especially in species exhibiting a nonfully mature intestine at birth. In mice, proliferation of the epithelium is extremely low until the second week after birth and is correlated with the number of goblet cells and mucus production [27, 84]. During the first 2 weeks, the intestinal epithelium developed specific strategies to tolerate the colonizing bacteria, such as secretion of neonate-specific antimicrobial peptides and constitutive active downregulation of the innate immune TLR4 pathway. By using mice with enterocyte-specific deletion of TLR4, Sodhi et al. demonstrated that TLR4 signaling prevents goblet cells differentiation by inhibiting Notch signaling, independently of the microbiota [85]. These findings might explain the low number of goblet and the low production of mucus during the 2 first weeks after birth, since the TLR4 signaling pathway is downregulated during this period [27]. Moreover, soluble factors contained in maternal milk also contribute to the protection of the neonates during breast feeding time and will be discussed later on. Of note, human milk contains mucin 1 and 4 which can bind pathogenic bacteria such as *Salmonella *Typhimurium and by competition with the host immune receptors, inhibit the invasion of epithelial cells [86]. Also, human milk oligosaccharides favor the selection of specific bacteria such as *Bifidobacterium infantis* which are able to consume them *via* mucus-utilization pathways, facilitating milk and solid food digestion [87].

Soluble factors are also released by mucosal cells into the forming mucus layer and reinforce the protection provided since they are jammed in a gradient manner in the mucus matrix. Apart from antimicrobial peptides, some secreted enzymes modify microbial ligands and thus prevent innate immune recognition and activation by the host. Among them, the intestinal alkaline phosphatase (IAP), contained in enriched vesicles released by epithelial cells, impairs LPS recognition by dephosphorylating LPS molecules and limits bacterial growth [88]. Likewise, the amidase peptidoglycan recognition protein-2 (Pglyrp-2) secreted by intraepithelial lymphocytes cleaves the muramyl dipeptide of the peptidoglycan, which impairs the recognition by the immune intracellular receptor Nod2 [89].

### 3.3. Antimicrobial Peptides Regulate Commensal Flora and Protect against Pathogens

Among the secreted molecules involved in the establishment of tolerance and in homeostasis, antimicrobial peptides play an important role. In addition to their bactericidal effects, antimicrobial peptides exert immunomodulatory functions, such as proinflammatory and chemoattractive activities, wound healing activation, and dendritic cell responses modulation [90]. In mammals, these ancient gene-encoded antibiotics are divided in two families: Defensins and Cathelicidins. *α*- and *β*-defensins consist of about 30 amino acids forming a triple-strand *β*-sheet structure with three intramolecular disulfide bonds. Defensins exhibit a broad range of bactericidal activity against Gram-positive and Gram-negative bacteria and also against fungi, viruses, and protozoa. Since they are highly cationic, they interact with the negatively charged phospholipids of the outer membrane of bacteria to disrupt the membrane integrity [91]. In the gut, Paneth cells, located in the crypt of the small intestine, produce constitutively high amount of *α*-defensins, human *α*-defensin (HD) 5, and HD6 in humans and more than 20 (also named cryptdins) in mice [92]. In mice, Paneth cells also produce another related family of antimicrobial peptides, the cryptdin-related sequence (CRS) peptides [93]. *α*-defensins secretion counts for 70% of the secreted bactericidal activity in Paneth cells. On the other hand, *β*-defensin proteins are expressed in the colon, although mRNAs have been detected in the small intestine. *β*-Defensins are regulated on the transcriptional level after innate immune activation. Conversely, *α*-defensins are posttranscriptionally regulated by proteolytic cleavage by the matrix metalloproteinase 7 (MMP7 or matrilysin) in mice and by the endoprotease trypsin after secretion in humans. Notably, MMP7-deficient mice exhibit alterations in the composition of the microbiota [94]. They also have been shown to be more susceptible to *Salmonella *Typhimurium infections, oppositely to humanized mice expressing HD5 which are more resistant [95, 96]. Studies of the expression of defensins during development show some discrepancies, probably due to the use of different experimental approaches and techniques (mRNA versus protein levels, use of oligonucleotide probes detecting several members of this conserved family, etc.). Nevertheless, it seems that expression of *α*-defensins 4 and 5 in mice is microbiota dependent, since germ-free mice exhibit a reduced level [97]. During postnatal development, it has been also noticed that *α*-defensins 1,3, and 6 exhibit a gradual increase whereas *α*-defensins 2 and 5 exhibit a rapid increase correlated with the appearance of crypts and Paneth cells after 2 weeks.

Cathelicidins are secreted in the gut by a variety of cell types, including neutrophils, mast cells, and epithelial cells. Mature cathelicidins result from the proteolysis of the C terminus of cathelin-domain-containing protein precursors, hCAP18 in human and CRAMP in mice. In cattle and pig, the diversity of this family of peptide is much more diverse. As defensins, the antimicrobial activity of cathelicidins is related to their cationic amphipathic properties, but they differ in their structure since they form *α*-helical or *β*-hairpin structures. Strikingly, cathelicidins have been shown to play a major role in the establishment of tolerance in neonates. Especially in mice, CRAMP is highly expressed at birth and during the first 2 weeks of life, independently of the enteric microbiota. The expression gradually disappears with the formation of the crypts and appearance of Paneth cells secreting *α*-defensins, which then seem to take over the major antimicrobial activity in the small intestine [98]. Of note, similar changes in the composition of antimicrobial peptides and in bactericidal activity during the postnatal period in humans have been detected [99]. It also has been shown that CRAMP-deficient neonates are more susceptible to *Listeria monocytogenes* infections [98]. The eventual role of CRAMP in the establishment and selection of the enteric microflora still remains to be investigated. 

At a lower extent, Paneth cells also secrete antimicrobial proteins, such as lysozyme P, secretory phospholipase A2, and the recently discovered C-type lectins Reg3*β* and Reg3*γ* [92]. Interestingly, it has been recently shown that, during the initial colonization of the gut, Reg3*β* and Reg3*γ* production known to be secreted is not only restricted to Paneth cells and absorptive enterocytes, since mRNAs were detected in goblet cells of small intestine and proximal colon between day 14 and 28 [100]. Recently, the active role of enterocytes in the control of bacterial load at the mucosal surface has been demonstrated by the fact that they produce Reg3*γ* through a pathway involving the interleukin (IL)-1R and TLR adaptor molecule Myd88 [101]. This production is at least in part supported by an intrinsic regulatory loop mediated by interleukin-22 (IL-22)-producing ROR*γ*t^+^ NKp46^+^ lymphocytes [102]. Reg3*γ* is essential to keep a 50 *μ*m bacteria-free zone above the small intestine epithelial surface and the antimicrobial effect is related to the capacity of specifically targeting native peptidoglycans on bacterial surfaces [103]. Specific deletion of Myd88 in intestinal epithelial cells results in increased number of mucus-associated bacteria, translocation of bacteria, decrease of the expression of Reg3*γ* as well as MUC2, and differences in the composition of microbiota [104]. Myd88-dependent expression of Reg3*γ* is also particularly important against Gram-positive bacterial infections, such as *Listeria monocytogenes* [105].

### 3.4. Maternal and Soluble Factors Participating to the Establishment of Tolerance in the Gut

Immunological priming can start prenatally, and maternal immune-active components derived from the placenta can influence the development of the gut immune system [34]. Secretory antibodies, such as IgG, are mainly transferred *via* the placenta in human and mice during the prenatal period [106]. At birth, the immaturity of the gut renders the neonate particularly exposed to microbes, and additionally to all the mechanisms previously cited in this review, maternal factors transmitted to the neonate through breast milk bring supplementary immunoprotection and help for the development of the immune system. Of note, amniotic fluid contains similar components compared to colostrum and has been shown to favor immune tolerance towards colonizing microflora, and administration to preterm pigs delivered by caesarian-section is protective against necrotizing enterocolitis [107]. Human milk is composed of 40 g/L lipids, 8 g/L proteins, 70 g/L lactose, and 5–15 g/L oligosaccharides. Colostrum and early breast milk contain large amount of IgA, immune cells such as neutrophils, macrophages, colostral corpuscules and lymphocytes, as well as soluble mediators such as cytokines (interleukins, INF-*γ*, TGF-*β*, etc.), hormones and growth factors (insulin, EGF, VEGF, CSF, etc.), nonspecific immune factors (oligosaccharides, lactoferrin, lysozyme, etc.) and even certain microRNAs [108–111]. In humans, a breast-fed infant consumes around 10^8^ immune cells per day, consisting of 55–60% macrophages, 30–40% neutrophils, and 5–10% lymphocytes. Maternal macrophages persist in the lumen of the neonate's gut during the first postnatal week and have even been found in the systemic circulation [112]. Also, maternal milk participates to the maturation of adaptive immune system since microRNAs associated with T-cell and B-cell differentiation have been detected [111]. As pointed before, analyses of the intestinal microbes of breast-fed human infants revealed that maternal milk also plays a role in shaping the microbiota. The breast-fed neonate is provided with 0.25–0.5 grams per day of secretory IgA antibodies *via* absorption of maternal milk. Maternal IgA restrict immune activation and microbial attachment by binding nutritional and microbial antigens. The appearance of IgA secreted by the neonate is correlated with weaning and plasma cells maturation. Of note, the specific deletion of Myd88 in intestinal epithelial cells induces a downregulation of polymeric immunoglobulin receptor, the epithelial IgA transporter, underlying the importance of Myd88 signaling in gut homeostasis [104]. Lactoferrin limits the pool of free iron and suppresses bacterial growth. Interestingly, miR-584 has been shown to induce the expression of the lactoferrin receptor in epithelial cells during the neonatal period [113]. The presence of oligosaccharides that can be utilized by specific bacteria such as *Bifidobacterium longum* spp. *infantis* also influences the composition of the neonatal microflora [114]. Interestingly, functional similarities between mammalian milk and crop “milk” produced by pigeons, flamingos, and emperor penguins to feed their young have been shown not only at the nutrition level, but also for the establishment of the immune system of the neonate and the maturation of the microbiota [115], which is particularly compelling from an evolutionary point of view. Thus, maternal milk contains factors to help the neonate to establish the microflora and to fight against pathogens. On the other hand, it also has been shown that it contains living bacteria (<3 log cfu/mL) and a range of bacterial components such as bacterial DNA [116]. Indeed, bacterial translocation from the mouse gut is increased during pregnancy and lactation, and bacterially loaded dendritic cells in the milk are thought to contribute to neonatal immune imprinting [117]. 

## 4. Neonatal Innate Immune Response, Infections, and Sepsis

### 4.1. Immune Stimulation, Epithelial Barrier Disruption, and Alteration of Microbiota

Proper development of immune tolerance is necessary for the maintenance of gut homeostasis and an efficient response against pathogens. Dysregulations of the mechanisms involved cannot only lead to inappropriate intestinal inflammation against microbiota such as inflammatory bowel diseases in some cases, but can also increase the susceptibility to bacterial infections and lead to neonatal sepsis [118]. In both human and mice, the immune response towards infections in the neonate is generally reduced compared to the adult response. Defects in mucosal immunity or even a response to infectious challenge can result in a dysbiosis, characterized by an altered commensal colonization of the gut. The disruption is usually a transient phenomenon, which is solved with the resolution of the infection and characterized by the return of the changed microbiota to baseline. However, some pathogens cleverly exploit host immunity to favor their invasion, such as *Salmonella *Typhimurium, *Citrobacter rodentium,* or *Campylobacter jejuni* [119]. The host response drives the disruption of the microbiota which enhances pathogen colonization and persistence, suggesting that host innate responses select for a characteristic microbiota composition. 

Pathogenic bacteria also developed species-specific mechanisms to cross the epithelial barrier though their interaction with host cell receptors. For example, *Salmonella *Typhimurium utilizes the receptor for epidermal growth factor (EGF) of epithelial cells, and the entry of the bacteria is coincident with tyrosine phosphorylation of the receptor. Also, both *Salmonella *Typhimurium and EGF induce patterns of host tyrosine phosphorylations that are remarkably similar [120]. *Salmonella *Typhimurium has also developed innate immune evasion mechanism such as O-antigen expression during apical intestinal epithelial invasion which delays the recognition of LPS by TLR4 [121]. In contrast, *Listeria monocytogenes* enters the enterocyte by using a zipper mechanism [122]. The mechanisms used by invasive *Escherichia coli* are different, and their capacity to inhibit NF-*κ*B activity allows them to damp the inflammatory response of the host [123]. Inflammation leads to production of nitric oxide, which is known to alter expression and localization of the tight protein zonulin ZO-1, ZO-2, ZO-3, and occludin, and increases epithelium permeability which favors bacterial translocation [124]. As pointed before, bacterial translocation is also observed under physiological conditions, since systemic bacterial DNA has been detected in healthy volunteers, suggesting a role for bacterial translocation in the development of the immune system [125, 126].

The immaturity of the neonatal immune system explains the age-dependent differences of the immune responses against pathogens as well as the susceptibility to different type of infections. For example, newborns are highly susceptible to *Shigella flexneri*, the causative agent of human bacillary dysentery, due to the lack of Paneth cells during early postnatal development. Also, MMP7-deficient mice show an increased inflammation and higher bacterial load after oral infection compared to wild type [127]. Similarly, the susceptibility to rotavirus is restricted to children under the age of 6 in human and is highest in between day 3 and 11 in mice. Interestingly, an upregulation during infancy of TLR3 expression on intestinal epithelial cells, which are the prime target of rotavirus, has been observed. This increase might contribute to the age-dependent susceptibility to rotavirus infection [62, 128]. Also, neonates have been shown to be more prone to *Salmonella *Typhimurium infections, correlating with an age-dependent increase of INF-*γ*, important for epithelial defense against intracellular pathogens [129]. 

### 4.2. Microbial Pathogenesis of Neonatal Sepsis

Neonatal sepsis (NS) is a major cause of morbidity and mortality among newborn infants, occurring in 1 to 10 per 1000 newborns, with a mortality rate of 15 to 50% [130, 131]. More than 10% of the neonates develop an infection during the first month of their life [132]. Despite the fact that symptoms are the same as an adult sepsis, the immune response of the neonate during NS is different. Due to the fact that the adaptive immune system of the neonate is not mature, the response induced is controlled by the innate immune system [133, 134]. Besides, no association was found between the TNF-*α*-308 G/A polymorphism blood culture-proven sepsis in very low birth weight infants, whereas the TNF-*α*-308 A allele is associated with higher sepsis in adult [135]. NS is clinically associated with a systemic infection during the 4 first weeks after birth, which can lead to pneumonia or meningitis. According to the time of symptoms appearance, NS is considered as early-onset neonatal sepsis (EONS) during the first 72 hours after birth, or late-onset neonatal sepsis (LONS) afterwards. This distinction largely contributed to improve the diagnostics and the treatment of this pathology, particularly because of the identification of the causative microorganism which varies depending on the age of the infant and also because of the origin of the infection depending on the time of appearance. 

A number of pathogens have been associated with NS and the predominant agents are bacterial, but viruses including herpes simplex and enteroviruses have also been associated with fulminant neonatal sepsis with high mortality [136, 137]. Causative agents for EONS are mostly microorganisms colonizing the maternal genital tract [138]. The most frequent microorganisms involved in EONS are Coagulase-negative *staphylococci* (CoNS), Group B *streptococci* (GBS), *Escherichia coli*, *Listeria monocytogenes*, and *Haemophilus influenzae* [138–140]. LONS are mostly caused by microorganisms from the external environment, often carried by care staff or by horizontal transmission [141]. The most frequent microorganisms involved in LONS are GBS, CoNS, and *Enterobacteriaceae*, including *Escherichia coli*, *Klebsiella pneumoniae*, and *Acinetobacter baumannii *[138, 142]. 

CoNS are responsible for 50% of LONS and more than half of very low birth weight infants are infected with CoNS, unlike full-term infants [143]. The recognition of CoNS by the innate immune system may have serious implications for preterm infants, since, for example, the expression of TLR4, although similar on term neonatal and adult monocytes [144], is significantly reduced in preterm infants [145]. The formation of biofilms by CoNS is essential for the pathogenicity of the bacteria by protecting themselves against host defense [146]. The polysaccharide intercellular adhesin (restricted to a subpopulation of *Staphylococci epidermidis*) and the poly-g-DL-glutamic acid (ubiquitous among *Staphylococci epidermidis* strains) protect the bacteria against cathelicidin and human *β*-defensin 3 [147, 148]. Moreover, the immaturity of the neonatal complement system impairs the capacity of neonates to fight against biofilm-associated *Staphylococci epidermidis* infections [149]. Interestingly, a study, dealing with the effects of lactoferrin with antibiotics commonly used in neonatal practice against CoNS, shows a synergic action, demonstrating that lactoferrin may be a promising agent to improve treatments of NS caused by CoNS [150]. 

If GBS is a component of the normal mucosal flora, in contrast, invasive GBS disease constitutes a rare event for which neonates present an increased risk. Neonates respond with a powerful inflammatory cytokine response to GBS and exhibit at the same time several deficiencies of components of the clearance mechanisms [151, 152]. Host factors underlying postnatal maturation and directly influencing elimination of GBS are likely to contribute to GBS sepsis in newborns. Extracellular GBS lipoproteins are known to interact with TLR2/6 and thereby contribute to GBS sepsis pathogenesis, and Myd88-dependent signaling is essential in innate immune response against GBS. Also, type I interferons (IFNs) and IFN-*γ*, IL-6, IL-12, and IL-18 contribute significantly to the course of GBS neonatal sepsis [153, 154]. The recognition of GBS and other Gram-positive bacteria by macrophages and monocytes relies on bacterial single-stranded RNA and phagocytosis induced NO in a Myd88 and UNC-93B-dependent manner but independently of known nucleotide-sensing TLRs [155, 156]. Recently, the activation of NLRP3 inflammasome, leading to production of IL-1*β* and IL-18, was also shown to be involved in host defenses against this pathogen [157].

*Listeria monocytogenes* represents an opportunistic pathogen which mainly infects immunocompromised patients, pregnant woman, elderly persons, and neonates [158]. In neonatal infections, *Listeria monocytogenes* can be transmitted from mother to child *in utero* or during vaginal delivery. In a neonatal mouse model, neonatal mice overproduce IL-10 during infection, which is known to trigger a detrimental effect [159]. Indeed, IL-10 blockade in neonates is protective during both early and late infection, whereas this effect is only observed at early stage in adult mice [160]. As pointed before, the cathelicidin CRAMP is highly expressed during the neonatal period and plays a prominent role in the protection of the newborn against pathogenic enteric bacteria, and particularly against *Listeria monocytogenes* [98]. Activation of the PI3 kinase and Rac1 *via* a TLR2-MyD88-dependent pathway facilitates the phagocytosis of *Listeria monocytogenes* by murine macrophages. In intestinal epithelial cells, *Listeria monocytogenes* is recognized by immune receptors such as Nod2 and Ipaf, and the NADPH oxidase (Nox) 4-dependent production of reactive oxygen species (ROS) allows horizontal intercellular communication. This mechanism favors the amplification of the immune response against bacteria [161].

Some recent studies have highlighted a significant reduction in GBS EONS with the increased use of prophylactic antibiotics, leading to an increase of rates of non-GBS infection and particularly an increase in EONS caused by *Escherichia coli* [140]. Enteropathogenic *Escherichia coli* (EPEC) destroys intestinal microvilli and suppresses phagocytosis to facilitate efficient infection [162]. The bacterial protein Hek, which promotes adherence to and invasion into cultured epithelial cells, has a key role in the transcytosis of *Escherichia coli* across the intestinal epithelial barrier [163]. Moreover, gut barrier dysfunction was recently shown to be mediated by an increase of HMGB1 following LPS administration, supporting the deleterious effect of *Escherichia coli* on bacterial translocation and potentially sepsis [164]. In a murine model of enterotoxigenic *Escherichia coli* (ETEC) infection, pretreatment with lactoferrin led to nearly full protection of gut-associated tissue, intact microvilli and decreases of activated cells [165]. Lactoferrin has also an inhibitory effect on the adherence of ETEC to epithelial cells [166]. 

### 4.3. Factors Involved in the Pathogenesis of Neonatal Sepsis

Commonly, associated risk factors to NS are maternal and environmental exposure, immune status, as well as the weight of the neonate at birth, and the time of the gestation period, making preterm and very low birth weight infants (<1500 g at birth) particularly susceptible [140]. The integrity of the intestinal barrier is a must to prevent the dissemination of microorganisms in the systemic compartment. Moreover, the level of permeability of the gut plays a key role in the pathogenesis of inflammatory pathologies such as ischemia-reperfusion or necrotizing enterocolitis, another inflammatory disorder leading to necrosis of the gut in neonates and particularly preterm infants [38, 167, 168]. Of note, an association between *Pseudomonas aeruginosa* sepsis and necrotizing enterocolitis has been shown [169]. Disruption of the neonatal barrier can be due to antibiotic treatment, hypoxia, or remote infection [170, 171]. 

Dysbiosis of the intestinal microbiota also predisposes the intestine of neonates to inflammation. Indeed, many operational taxonomic units, which are frequently detected in healthy controls, were not detected in LONS cases [172]. This data suggests that a lack of colonization by various normal or nonpathogenic bacteria, rather than the presence of a pathogen, might increase the risk of LONS. Based on this finding, it has been proposed that a delay in colonization by proteobacteria, which is normal and immunologically well tolerated during the initial weeks of microbiota development, might result in an excessive immune response that compromises the integrity of the mucosal barrier, thereby allowing translocation of bacteria into the circulation resulting in LONS and extensive inflammation. Moreover, neonates developing sepsis present a low microbial diversity compared to healthy infants [173]. The specific mechanisms linking the intestinal microbial changes to sepsis remain unclear, but recent studies favor the idea that disruption of the normal intestinal microbiota and induction of the inflammasome are potential mechanisms [174, 175]. Moreover, the number of *Bifidobacteria*,known to colonize the healthy newborn intestine soon after birth and likely contribute to normal intestinal development, is lower in LONS infants compared to healthy controls [172, 176].

A protective effect of human breast milk against infection and sepsis/meningitis in very low birth weight as well as in full-term infants has been described [177]. The large amount of glycans in the milk seems to protect the neonate from many bacterial, viral, fungal, and other pathogens [178, 179]. Secretory IgA of the milk is known to inhibit the association of bacteria with the gut mucosa and reduce bacterial penetration in the gut. In neonates, IgA supplementation is known to avoid bacterial translocation by enhancing gut mucosal barrier function and therefore neonatal gut-origin sepsis [180, 181]. As pointed before, lactoferrin, which is present in human milk, is a component of innate immunity and has antimicrobial activity. In several *in vitro* and *in vivo* models, lactoferrin shows potent protective effect on infections with enteric microorganisms, such as *Staphylococci epidermidis*, *Escherichia coli*, and rotavirus [182–184]. Moreover, a recent study has shown the beneficial effects of oral lactoferrin prophylaxis for the prevention of sepsis and necrotizing enterocolitis in preterm infants [185].

The pathology of sepsis involves highly complex interactions between pathogens, immune response of the host, and multiple downstream events leading to organ dysfunction and death. Studies in twins suggest that genetic factors are also involved and contribute to variations in susceptibility to infections. Candidate genes have been suggested to play a role in the pathogenesis of sepsis [186, 187]. Several polymorphisms associated with neonatal sepsis have been identified in genes playing a role in host innate immunity: the phospholipase A2, the pattern recognition receptors TLR2 and TLR5, the anti-inflammatory cytokine IL-10, and the serum mannose-binding lectin (MBL) [188, 189]. Moreover, mutations of genes also involved in the innate immune system have been associated with sepsis in very low birth weight infants, such as CD14, TLR4, NOD2, IL-6, and MBL [190]. Identification of these genetic variations may allow the development of new diagnostic tools and more accurate predictors, as wells as the improvement of the classification of sepsis.

## 5. Conclusion

**Figure 2:**
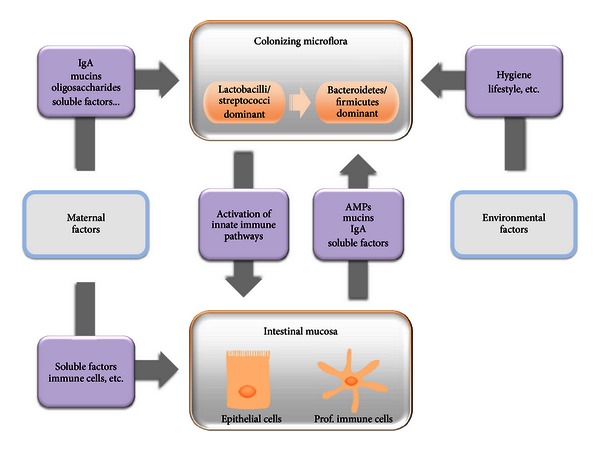
Factors involved in postnatal intestinal innate immune adaptation. The colonizing microflora and the intestinal mucosa interact in a two-way street to establish life-time tolerance and mutualism between each other. While bacteria activate innate immune pathways in host epithelial and immune cells inducing immune maturation and tolerance, mucosal cells produce factors (antimicrobial peptides AMPs, mucins, immunoglobulin A IgA, etc.) to control the number and the composition of bacteria. Microflora is also influenced by environmental factors (hygiene, lifestyle, etc.). Maternal factors, such as IgA, mucins, oligosaccharides, or other soluble factors, can modulate the microflora, and contribute to improve host immune defense and maturation (maternal immune cells, soluble factors, etc.).

**Figure 3:**
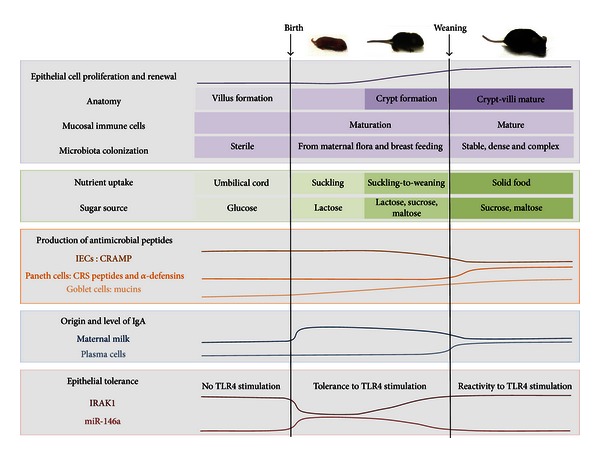
Summary of changes taking place in the intestine during the fetal period, neonatal period, and adult period. Please refer to the text for details. IECs: intestinal epithelial cells: CRAMP: cathelin-related antimicrobial peptide; CRS: cryptdin-related sequence; IgA: immunoglobulin A; TLR: toll-like receptor; IRAK1: interleukin-1 receptor-associated kinase 1; miR-146a: microRNA 146a.

Outside the uterus, the neonate, which is a unique host immunologically, is exposed to environmental microbes and endotoxins. Immune adaptation of the gut to extrauterine life is extremely important and complex (Figures 2 and 3). External factors such as breast feeding, environment, or delivery mode, as well as genetic factors influence this process which is largely microbiota dependent. The microbiota is an essential complex and multifunction ecosystem which functions as an extra organ, shaping the immune system of the host, and is by itself sculpted by the host immunity. Indeed, interactions between microbiota/microbes components and intestinal epithelial cells largely drive the establishment of homeostasis during the neonatal period and also allow its maintenance during adult life. Pattern recognition receptors, seen initially as the first sentinel in the fight against microbes, play also obviously a major role in the tolerogenic response, and their signaling needs to be tightly fine-tuned spatially and temporally. Since the same receptors can have both beneficial and deleterious effects, the understanding of these aspects requires sustained efforts and probably more work on the animal models used currently, as well as on the tools to study the microbiota. Breaking the balance between all the players of the immune tolerance can induce inflammatory diseases and an increased susceptibility to infections. In neonates, the failure to establish immune tolerance leads to important mortality and morbidity and is particularly crucial for the survival of premature infants that are even more susceptible to infections and sepsis. Despite major advances in neonatal intensive care, infections and sepsis continue to be an important cause of death. A better understanding of the key mechanisms involved to establish and keep the balance between all the players of the immune tolerance will allow the discovery of efficient therapeutic and prophylactic tools to improve the medical care of those infants.
